# Clinical Lectures on the Diseases of Women and Children

**Published:** 1855-09

**Authors:** 


ART. VII.— *Clinical Lectures on the Diseases of Women and Children.* By Gunning S. Bedford, A. M., M. D., Professor of Obstetrics, Diseases of Women and Children and Clinical Midwifery in the University of New York. New York: S. S. & Wm. Wood, 261 Pearl St. 1855.

A large experience, and a long service as teacher in the specialties named in the title-page, have conferred on Prof. Bedford the means of framing a useful work upon this department of practical medicine. At the outset, however, we are compelled to find fault with the vehicle of communication.

Clinical lectures derive their value from the actual presence of disease. The oral instruction at the bedside is no dull didactic prelection, requiring close attention and concentration of mind to follow, it is a free off-hand commentary on what passes at the moment beneath the eye, the touch, the smell, the hearing. All the senses lend their aid to the instructor, and give to him in a certain sense, a secondary importance. He is not now the historian, the philosopher of disease—he is the guide, the *cicerone* who points out objects of interest and importance.

To our sense a written out, printed, clinical lecture, read by lamplight five hundred miles away from its place of delivery, is about as instructive as would be the well conned speech of the cicerone of Naples, delivered on the corner of streets yet unnamed, in some western embryo city. It is Hamlet, with the part of Hamlet omitted; a clinical lecture without the clinic, demonstrative teaching without the demonstration.

So with these lectures of Dr. Bedford. The presence of disease, the one fact which gave his teachings their life and interest, is wanting, and the imagination painfully endeavors to fill the deficiency by conjuring up for the minds eye the appearance of the patient. She was Irish, of course; probably pock-marked; short and thick, perhaps; maybe gaunt and skinny. Had she black hair or red? did they bring her into the room, or did she walk in with the tramp of a grenadier? All these, and a thousand other questions, arise to confound all hopes of that intelligent appreciation of this individual case, without which it is not worth while to inquire about the treatment.

These are radical faults which will certainly destroy the value of any book constructed on the plan of a verbatim report of clinical remarks. We do not mean to assert that the lectures at the time they were delivered, were not extremely valuable to the students who attended them. He must have been stupid, indeed, who could not profit by the exhibition of such an array of disease, but one cannot keep his dinner and eat it at the same time, and once delivered, Dr. Bedford's remarks were spent powder.

It seems to us that Dr. Bedford has fallen into a grievous error in supposing that his teachings could maintain their value when stripped of both accessories and essentials. He was, doubtless, to some extent conscious of this, but admiring followers had reported his lectures, the American Lancet had printed them, and to make a book he had but to hand over a file of the Lancet to his publishers. These circumstances made it easy to get up the volume, and the advertisement of his clinic and school which would result from its publication, would make it profitable. The temptation was a strong one, as neither labor nor risk were to be met; but, alas, *good* books are not so easily put together.

The only manner in which clinical instruction can be made profitable to the *reading* public, is by making a series of cases the subject of classification and philosophical induction. Two months ago we published a clinical lecture by Dr. Bedford's colleague, Prof. Metcalfe, in which this end was admirably accomplished, and the cases were as interesting to the reader as to the witness of them. But here it was Dr. Metcalfe who was the essential thing, the cases were secondary, tributary to an idea, and worthless except from the light thrown by the teacher from them to the subject.

Another and a serious objection to this style of book manufacture, is the entire want of arrangement or classification of cases. Take, for instance, the programme of a single lecture, selected at random. Its multifold subjects succeed one another in the following order: "Mucous discharge from vagina—Vaginal discharges generally—Importance of accurate diagnosis—Whites—Intestinal worms—Origin of worms—Vascular tumor of the meatus urinarius—Ulcerative carcinoma of the cervix uteri—Human credulity—Heartless exactions of the quack—Suppression of the menses—Cholera morbus."

This is a curious *olla podrida*, not wanting in garlic to give it a relish. But, as might be expected, a book so arranged offers few inducements to the student. Take up any one subject and you find it scattered through the thirty chapters indiscriminately, in juxtaposition with all manner of irrelevant odds and ends. As might be expected, some subjects get little notice, while others have an undue share. Leucorrhœa receives two pages of consideration only, out of five hundred and fifty.

We have been betrayed into a wider range of comment than we had anticipated. Thus far our objections have referred to manner rather than matter, to the faulty and imperfect style in which Prof. Bedford has chosen to bring himself before the profession, rather than to what we may consider errors of doctrine. And we may confess at once that we feel hardly at liberty to set up our small authority against a teacher so widely known. More than this, there is very little in the way of doctrine or pathology to consider. In his anxiety to make a thoroughly practical work, Prof. Bedford has confined his remarks to the causation, aspect, and treatment of disease. In all these subjects he is necessarily somewhat superficial, while in pathology, especially, he is remarkably loose and incorrect.

This is, probably, the result of the style of teaching. Few clinical lecturers can be always prepared to stand by and defend all the dogmata hurridly and inconsiderately thrown out at the bedside. The greater the necessity, then, of care in publishing. Doubtless in his formal didactic lectures before his class, Dr. Bedford was not wanting in these points—at any rate we are content to assume this as a fact, and so avoid what might otherwise be our duty as a critic, viz: to examine carefully the scholarship and accuracy of Dr. Bedford's teachings.

We do not base any opinion as to Dr. Bedford's talents, as an author, on this volume. We should be very sorry to judge him by it alone, in the hope that in a book constructed more in accordance with the necessities of scientific teaching, and not so utterly wrong in conception as the one under review, he might do far greater honor to himself and to the science in which he has so long been a distinguished teacher.

For sale by PHINNEY & Co.

